# Beyond the barrier: Targeting blood–brain interactions for neuroprotection and repair

**DOI:** 10.4103/NRR.NRR-D-25-00723

**Published:** 2025-09-29

**Authors:** Anddre O. Valdivia, Caroline Brandt, Mark A. Petersen

**Affiliations:** Department of Pediatrics, University of California San Francisco, San Francisco, CA, USA

The central nervous system (CNS) does not function in isolation—it engages in continuous molecular dialogue with the vascular and immune systems. Traditionally, the blood-brain barrier (BBB) was portrayed solely as an impermeable wall, safeguarding the CNS by excluding blood–derived molecules and circulating cells. However, this view has evolved. The BBB is now recognized as a dynamic interface that selectively regulates the exchange of signals, cells, and molecules between the bloodstream and the CNS to maintain a homeostatic neurovascular environment. “BBB breakdown” thus refers not only to the physical deterioration of cell-to-cell junctions but also to alterations in transport mechanisms and transcytotic pathways that increase vascular permeability. When this finely tuned balance is disrupted, the influx of neurotoxic blood proteins and immune cells transforms the stable neurovascular niche into a proinflammatory environment, triggering processes that initiate and accelerate the progression of neurological diseases (Akassoglou et al., 2024). These blood-derived factors act as upstream triggers of neuroinflammation, activating microglia, the resident immune cells of the brain, which further damage the BBB and the surrounding brain tissue. This bidirectional interplay reinforces a pathological cycle of inflammation and barrier compromise, propagating neural injury in a spectrum of CNS diseases from development through aging. Among these factors, fibrinogen has emerged as a central molecular orchestrator of neurovascular pathology (Petersen et al., 2018). Beyond its classical role in coagulation, growing evidence shows that fibrinogen is a key driver of neuroinflammatory responses and a regulator of CNS stem and progenitor cells, suppressing regenerative signaling pathways and impairing both brain development and repair (Nath et al., 2024; Ryu et al., 2024; Weaver et al., 2024). These insights shift our understanding of blood–brain interactions from secondary consequences of neurological disease to active barriers to regeneration, highlighting new targets for therapeutic intervention to promote CNS repair.

**Blood–brain interactions in developmental brain injury:** CNS hemorrhage is the most common brain injury in preterm infants and a major cause of long-term neurodevelopmental impairment. One of the earliest and most affected regions is the germinal matrix (GM), a highly angiogenic, progenitor-rich zone containing radial glia, immature neuronal and glial progenitors, oligodendrocyte progenitor cells (OPCs), microglia, and vascular cells (Chen et al., 2025). The dense angiogenic vasculature of the GM near the ventricular surface is particularly susceptible to rupture. Germinal matrix hemorrhage (GMH), which affects up to 40% of extremely preterm infants, initiates a cascade of neuroinflammation, impaired neurogenesis, and disrupted myelination, altering the development of both the forebrain and cerebellum. Affected infants face high rates of cerebral palsy, cognitive impairment, and neurodevelopmental disorders (Chen et al., 2025). Currently, no specific treatments exist to prevent the developmental impairments associated with GMH.

Recent findings have deepened our understanding of how interactions at the blood-brain-immune interface contribute to the vulnerability of the GM to hemorrhage (Chen et al., 2024). In the developing forebrain, CD45^+^ macrophages and microglia play a critical regulatory role in shaping the immature vasculature. Under normal conditions, these cells support angiogenesis and vessel stability. However, in the context of GMH, immune cell populations shift toward a pro-inflammatory phenotype with activated neutrophils and monocytes releasing factors such as azurocidin 1, elastase, and CXCL16. These molecules compromise the structural integrity of the vasculature by disrupting endothelial junctions and increasing permeability, creating a permissive environment for bleeding. This shift converts a normally supportive neurovascular niche into a destabilized, hemorrhage-prone landscape, positioning immune-driven vascular disruption as a primary initiator of GMH pathology.

The resulting breach in the BBB during inflammatory or hemorrhagic injury facilitates the entry of blood-derived factors, such as hemoglobin, iron, antibodies, complement, thrombin, and coagulation proteins, into the CNS, where they can exert potent effects on cellular signaling. Among these, fibrinogen has proven to be a key inhibitor of developmental and regenerative processes. In the neonatal cerebellum, fibrinogen increases microglial number and impairs sonic hedgehog (SHH) signaling, a pathway critical for granule neuron progenitor proliferation, indicating parallel effects on immune reactivity and neurodevelopment (Weaver et al., 2024). By suppressing SHH signaling and downstream gene expression, fibrinogen limits progenitor cell expansion and restricts cerebellar growth at a critical stage of maturation. Even low levels of fibrinogen in the extracellular matrix are sufficient to disrupt this mitogenic environment, underscoring the sensitivity of the cerebellum to blood-derived factors. Notably, genetic depletion of fibrinogen attenuates neuroinflammation, restores SHH signaling, and rescues cerebellar growth in neonatal mouse models of cerebellar injury. Thus, in the context of barrier disruption, fibrinogen acts as a molecular antagonist of developmental neurogenesis.

These findings converge on a unifying theme: reciprocal interactions between blood proteins, immune cells, and neural progenitors within a compromised neurovascular microenvironment can reprogram and disrupt developmental trajectories. Fibrinogen, in particular, exerts a dual role—amplifying innate immune responses while directly suppressing regenerative signaling in CNS stem and progenitor populations (Petersen et al., 2018). This illustrates how neurovascular injury and immune dysregulation synergize to derail neurodevelopment, affecting critical early structures like the GM as well as later-maturing regions such as the cerebellum.

**Neurovascular inflammation and the inhibition of neurorepair in the adult CNS:** While these mechanisms are particularly destructive during early brain development, similar processes persist, and often intensify, following injury in the mature CNS. In the adult brain, repair and regeneration rely on the tightly regulated activity of endogenous stem and progenitor cell populations, including neural stem/progenitor cells in the subventricular zone and OPCs distributed throughout white matter. However, these regenerative processes are highly susceptible to disruption by neuroinflammation and BBB compromise. As in the developing CNS, fibrinogen is at the center of this pathophysiological axis, where it actively impairs neurorepair through both immune-mediated and direct molecular mechanisms (Petersen et al., 2017; Petersen et al., 2021; Nath et al., 2024; Ryu et al., 2024).

After cortical stroke, the subventricular zone, a major site of adult neurogenesis, undergoes a series of profound changes. Vascular permeability increases, allowing fibrinogen to accumulate within the stem cell environment, and resident microglia are rapidly activated, disrupting this delicate niche (Nath et al., 2024). This triggers an abnormal neurogenic response: neural stem/progenitor cells are activated and begin to proliferate, but subsequent lineage progression stalls. Intermediate progenitors exhibit cell-cycle arrest, and neuroblasts undergo increased apoptosis. Single-cell transcriptomics reveals that activated microglia form discrete subclusters with altered ligand-receptor signaling that suppresses neural stem/progenitor cell survival and differentiation. Importantly, interventions that either deplete microglia or restore niche homeostasis, such as fibrinogen depletion, rescue neuroblast survival and enhance the regenerative output of the subventricular zone (Nath et al., 2024). These findings emphasize the role of blood-immune-mediated niche dysfunction in limiting endogenous repair.

Beyond its immunomodulatory effects, fibrinogen also directly inhibits CNS progenitor cells in a cell-autonomous manner. In animal models that mimic multiple sclerosis or stroke, fibrinogen deposits in the perivascular extracellular matrix serve as an upstream trigger for bone morphogenetic protein (BMP) signaling in neural stem/progenitor cells and OPCs (Pous et al., 2020; Petersen et al., 2021). In demyelinated lesions, fibrinogen activates the activin A receptor type I (ACVR1) BMP receptor, which suppresses OPC differentiation into myelinating oligodendrocytes and instead promotes astrocytic fate, thereby impairing remyelination (Petersen et al., 2017). Pharmacologic depletion of fibrinogen or inhibition of BMP receptor signaling restores myelin regeneration, identifying this pathway as a critical checkpoint in the process of remyelination (Petersen et al., 2017, 2021).

Further linking fibrinogen to long-term neurological impairment, studies in the context of coronavirus disease 2019 (COVID-19) have shown that fibrin, the insoluble end-product of fibrinogen cleavage during coagulation activation, drives both systemic thrombo-inflammation and CNS pathology (Ryu et al., 2024). Through activation of the CD11b/CD18 integrin receptor on innate immune cells, fibrin is known to trigger proinflammatory and neurotoxic pathways that exacerbate vascular damage and sustain an inhibitory neurovascular environment (Petersen et al., 2018). Additionally, fibrin binds the severe acute respiratory syndrome coronavirus 2 (SARS-CoV-2) spike protein to form highly inflammatory, fibrinolysis-resistant clots that further promote reactive microgliosis and neuronal loss, even in the absence of active infection (Ryu et al., 2024). These pathogenic mechanisms reinforce the concept that fibrinogen is not a passive marker of injury, but an active regulator of inflammatory and regenerative responses in the brain.

Together, these findings shift our understanding of why the adult brain so often fails to repair itself. Molecular signals at a pathological blood-brain-immune interface drive neurodegeneration and inhibit neurorepair. Among these, fibrinogen emerges as a key disruptor that hijacks the healing process. It fuels neuroinflammation, pushes microglia into a toxic state, and shuts down the signaling pathways that neural and glial progenitor cells need to regenerate. What should be a window for recovery instead collapses into a deadlock, with the very systems meant to protect the brain turning against it. Yet within this disruption lies a therapeutic opportunity: by targeting fibrinogen and the broader blood-brain signaling pathways it controls, it may be possible to reawaken the brain’s innate capacity for regeneration and reopen the door to repair.

**Figure 1:**
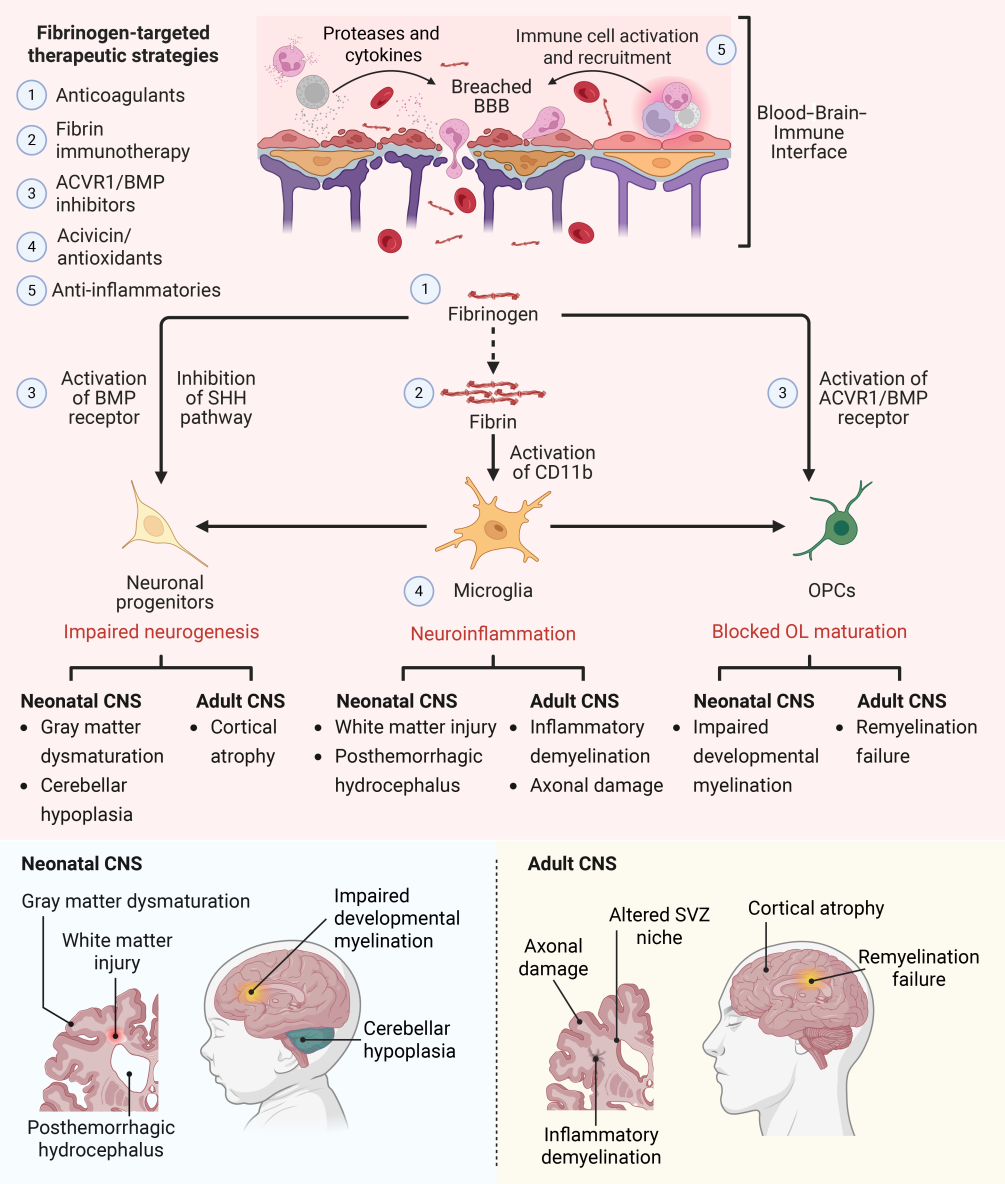
Neurovascular mechanisms and therapeutic targeting of fibrinogen at the blood-brain-immune interface. Blood–brain barrier (BBB) disruption permits the entry of blood-derived factors such as fibrinogen into the central nervous system (CNS), triggering a cascade of neuroinflammatory and anti-regenerative responses. Fibrin(ogen) contributes to neuroinflammation, impaired neurogenesis, and blocked oligodendrocyte (OL) maturation through multiple mechanisms: activation of CD11b on microglia, suppression of sonic hedgehog (SHH) signaling in neuronal progenitors, and induction of bone morphogenetic protein (BMP) signaling in oligodendrocyte progenitor cells (OPCs) and neuronal progenitors. This may drive neuropathology in neonatal (e.g., gray and white matter injury, posthemorrhagic hydrocephalus, impaired myelination, cerebellar hypoplasia) and adult (e.g., inflammatory demyelination, axonal damage, subventricular zone [SVZ] disruption, cortical atrophy, remyelination failure) CNS disease. Targeted therapeutic strategies—such as anticoagulants, selective fibrin immunotherapy, activin A receptor type I (ACVR1)/BMP inhibitors, antioxidants, and immunomodulators—aim to disrupt fibrin(ogen)-driven pathology, restore neurovascular homeostasis, and promote neurorepair across the lifespan. Created in BioRender. Petersen, M. (2025) https://BioRender.com/zvy9gel.

**Restoring balance at the blood–brain border:** As evidence accumulates that blood–derived signals, particularly fibrinogen, act as active disruptors of neural development and repair, therapeutic strategies are beginning to converge on the neurovascular interface as a promising site for intervention. Rather than focusing exclusively on downstream consequences of neuroinflammation or degeneration, these emerging approaches aim to intercept the apical molecular triggers that destabilize regenerative environment of the brain. The goal is to re-establish a permissive niche by targeting key interactions at the blood–brain–immune interface to attenuate pathological signaling and preserve homeostatic functions. Fibrinogen provides a clear model for this strategy—its central role in many neurological diseases is enabling the development of targeted interventions that neutralize its harmful CNS effects while sparing its essential systemic functions (**Figure 1**).

***Strategy 1 – Fibrinogen depletion:*** Preclinical studies using genetic and pharmacological fibrinogen depletion have demonstrated robust protective effects in models of developmental and adult CNS injury (Petersen et al., 2018; Weaver et al., 2024). Fibrinogen removal has been shown to reduce neuroinflammation, restore progenitor cell function, and enhance neurogenesis and remyelination across diverse disease models (Petersen et al., 2017; Nath et al., 2024; Weaver et al., 2024). However, complete fibrinogen depletion is not without risk. As a critical component of the coagulation cascade, systemic fibrinogen is essential for hemostasis, and its loss increases the potential for hemorrhage, which would be particularly problematic in populations already prone to bleeding, such as preterm infants or stroke patients. This limitation highlights the need for more selective approaches that preserve fibrinogen’s beneficial systemic functions while neutralizing its pathological effects in the CNS.

***Strategy 2 – Blocking pathogenic fibrinogen interactions:*** Rather than depleting fibrinogen entirely, recent efforts have focused on selectively inhibiting its deleterious interactions within the brain. A leading approach targets the cryptic fibrin epitope γ377–395, which is exposed upon conversion to fibrin and engages the CD11b/CD18 integrin receptor on innate immune cells. The monoclonal antibody 5B8 has been developed to specifically block this interaction, suppressing microglial activation, oxidative stress, and neurodegeneration without impairing coagulation (Kantor et al., 2023; Akassoglou et al., 2024). This strategy demonstrates how molecular precision can decouple the inflammatory and anti-regenerative roles of fibrinogen from its clotting functions, offering a safer and more targeted therapeutic profile. A high affinity, humanized version of the 5B8 antibody has now progressed to phase 1 clinical trials (Kantor et al., 2023).

***Strategy 3 – Modulating downstream signaling pathways:*** The impact of fibrinogen extends beyond immune activation to direct inhibition of progenitor cell signaling. In the neonatal cerebellum, fibrinogen suppresses SHH signaling and limits neurogenesis; in adult demyelinating lesions, it activates BMP signaling in OPCs, blocking oligodendrocyte differentiation and remyelination (Petersen et al., 2017; Weaver et al., 2024). Pharmacologic inhibition of these downstream pathways, such as BMP receptor antagonism with ACVR1 inhibitors, has been shown to restore regeneration in animal models, even in the continued presence of fibrinogen (Petersen et al., 2021). Furthermore, oxidative stress is a key mediator of fibrinogen-induced neurotoxicity. Targeting redox imbalance with antioxidants such as acivicin, an inhibitor of glutathione degradation, has shown promise in suppressing chronic neuroinflammation (Mendiola et al., 2020). These findings highlight the therapeutic value of targeting fibrinogen-induced signaling cascades that limit repair, without needing to completely eliminate the protein itself.

In conclusion, the recognition of blood-derived factors such as fibrinogen as active modulators of neurovascular injury, inflammation, and progenitor cell dysfunction marks a pivotal shift in our understanding of CNS injury and repair. Rather than focusing solely on downstream damage, emerging therapies target the upstream molecular disruptions at the blood-brain-immune interface, where vascular instability, immune activation, and regenerative failure intersect. In developmental brain injury, for example, fibrin-targeted immunotherapy could suppress blood-induced inflammation and oxidative damage while preserving essential clotting function—potentially limiting the progression of GMH. In parallel, small-molecule modulation of BMP and SHH signaling may help restore a regenerative environment at sites of neurovascular disruption, promoting neurogenesis and myelination. Furthermore, thrombo-inflammatory blood biomarkers may enable early identification of at-risk individuals, guide patient selection, and serve as dynamic tools for monitoring therapeutic response in clinical trials. Together, the development of fibrinogen-targeted therapies and blood-based biomarkers offers a blueprint for a broader pipeline to counteract the detrimental effects of neurovascular disruption in the CNS. This integrated approach holds great promise for advancing precision neurotherapeutics and reactivating the brain’s intrinsic capacity for repair.

*This work was supported by the National Institute of Neurological Disorders and Stroke of the National Institutes of Health under Award Number K02NS110973 and R01NS126498 (to MAP). The content is solely the responsibility of the authors and does not necessarily represent the official views of the National Institutes of Health.*

*MAP is an inventor on a pending patent related to fibrin. His interests are managed in accordance with the University of California San Francisco’s conflict of interest policies.*
